# Nonsurgical Management of a Papillary Fibroelastoma of the Aortic Valve

**DOI:** 10.1155/2021/4160793

**Published:** 2021-07-06

**Authors:** Antonino M. Grande, Nicoletta Castiglione, Adelaide Iervolino, Francesco Nappi, Antonio Fiore

**Affiliations:** ^1^Department of Cardiac Surgery IRCCS Fondazione Policlinico San Matteo, Pavia, Italy; ^2^Department of Cardiovascular Sciences, Fondazione Policlinico Universitario A. Gemelli IRCSS, Italy; ^3^Department of Cardiac Surgery, Centre Cardiologique du Nord de Saint-Denis, Paris, France; ^4^Department of Cardiac Surgery, Henri Mondor University Hospital, Assistance Publique-Hôpitaux de Paris, Créteil, France

## Abstract

We report the case of a 63-year-old woman who had an incidental echocardiographic diagnosis of papillary fibroelastoma (PFE) of the right coronary cusp of the aortic valve. The patient was informed about the embolic risk due to the pedunculated mass located on the aortic valve but she refused the proposed surgical removal. She was followed up yearly, and each follow-up included an echocardiographic evaluation of the mass. The lady is taking lysine acetylsalycilate 160 mg daily, and after more than 19 years later, she does not complain any symptoms or complications as a result of possible embolic episodes. If on one hand, our report is provocative for PFE nonsurgical management; on the other, we do believe that in symptomatic patients PFE located in the left heart chambers, the standard of care remains surgical excision after diagnosis. Anyway, our analysis shows that further data in this issue are needed in asymptomatic patients, and surgical indication should be proposed considering carefully the risk-benefit balance.

## 1. Introduction

Cardiac papillary fibroelastoma (PFE) represents 7.9% to 10% of all cardiac tumours and is the third most common primary benign cardiac tumour after myxoma (27%) and lipoma (10%) [1–4]. Before 1980, PFE was diagnosed as autoptic finding or incidentally at surgery [2, 3]. Echocardiography allows to diagnose most of these tumours antemortem with the possibility of curative surgical treatment. PFE mainly affects cardiac valves and as matter of fact represents more than 75% of valve tumours and occurs in almost all age groups, with a mean age of detection around 60 years, and its incidence is higher in men [5]. In addition, it is well known that PFE has the potential to determine embolic events that may jeopardize a patient's life. As a matter of fact, surgical removal of PFE is mandatory for all patients that have symptoms, but the treatment for those who are asymptomatic is still controversial.

## 2. Case Report

A 63-year-old woman was hospitalized in November 2001 in our department for surgical coronary revascularization. In 1998, she had an anterior acute myocardial infarction, and in January 1999, complaining exertion induced angina, she underwent coronary angiography and subsequently percutaneous transluminal coronary angioplasty of the left anterior descending (LAD) coronary artery. A year and a half later, she complained again exertional angina, and a repeated angiography showed a LAD occlusion and 90% stenosis of the first diagonal, while right and circumflex arteries showed a normal caliber; left ventricle ejection fraction measured by ventriculography was 50% and echocardiography showed only a mild mitral regurgitation. At that time, the lady underwent LAD grafting via a left anterior thoracotomy, with the left internal mammary artery (LIMA) as conduit. Two weeks later, she presented with recurrent exertional angina and coronary angiography showed occlusion of the LIMA graft. Reoperation was performed again via left anterior thoracotomy, and LAD bypass grafting was obtained using a tract of the saphenous vein. The cause of acute LIMA occlusion was due to technical issues at the level of the anastomosis. Postoperative course this time was uneventful, and the patient was transferred to rehabilitation department. Here, in January 2002, transthoracic echocardiography showed a small mobile mass on the right coronary cusp. The mass on the aortic valve was later confirmed by transesophageal echocardiography (TEE): a mobile, pedunculated, 0.8 cm round mass on the aortic side of the right coronary cusp of a trileaflet aortic valve, with an appearance most consistent with a PFE. She was afebrile and blood cultures were negative, ruling out endocarditis. Considering that we routinely use perioperative transesophageal echocardiography (TEE) during cardiac surgery, we may believe that PFE formed after the revascularization procedure.

**Figure 1:**
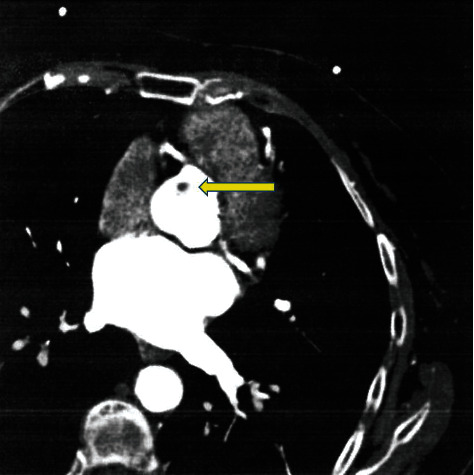
Computed tomography of the heart: the yellow arrow indicates the small round mass located on the right coronary cusp, 11 mm in diameter. Note the origin of the right and left coronary arteries.

Although the patient was adequately informed of the risk of stroke, systemic embolism, and the risk of myocardial infarct, she always refused surgery. Therefore, the patient was followed up at 12-month intervals, and each follow-up included an echocardiographic evaluation of PFE. She is under antiplatelet therapy with a daily 160 mg dose of lysine acetylsalycilate. In November 2011, a contrast computed tomography scan showed a mildly ill-defined, nonenhancing, hypodense nodular lesion, 11 mm in diameter, in the aortic root on the right coronary cusp of the aortic valve (Figure 1).

**Figure 2:**
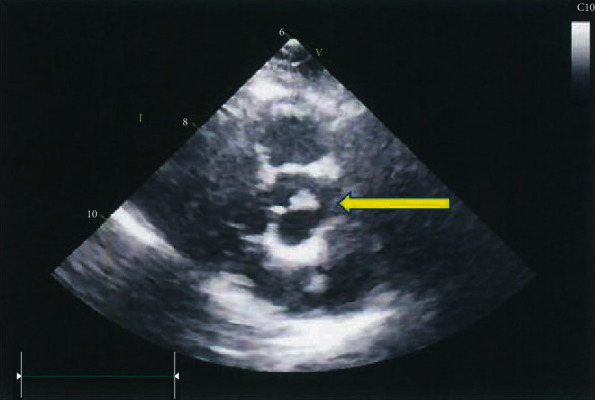
Transthoracic echocardiography: parasternal short axis shows a round mass, 9 mm in diameter (yellow arrow), on the aortic side of the right coronary cusp of the aortic valve.

In 2015, she had a mastectomy for breast cancer followed by chemotherapy. The patient after more than 19 years later did not complain any symptoms or complications as a result of possible embolic episodes. Over the years, the echocardiographic size of the mass did not significantly increase: in October 2017, PFE diameter was 9 mm as indicated in Figure 2.

## 3. Discussion

Erroneous diagnoses of a cardiac mass may lead to inappropriate treatment. There are several normal structures that may mimic a cardiac mass: the Eustachian valve, Chiari network, crista terminalis, pectinate muscles, moderator band, trabeculations, interatrial septal aneurysm, and lipomatous hypertrophy of the interatrial septum are some examples of normal structures that are frequently mistaken for pathologic entities [6, 7]. In the case of valvular mass, if valvular destruction or regurgitation is present, a vegetation rather than PFE should always be suspected.

Mutlu et al. [8] describe the case of a 63-year-old woman with an incidental echocardiographic 1 × 1 cm mass attached to the inferior wall of the left ventricle by a stalk, showing motion with cardiac cycle. The patient refused surgical removal, and she was kept on anticoagulation with warfarin and regularly followed up for 4 years without developing any symptoms or complications due to embolism. Küçükoglu et al. [9] reported a PFE located at the chorda of the anterior mitral leaflet in a 26-year-old woman complaining atypical chest pain: tumour diagnosis was made in 1988, and the patient was operated two years later but the attempt to remove the mass was unsuccessful. Therefore, the patient underwent conservative follow-up for the next 6 years, during which she complained palpitations and asthenia, until she went reoperation. At surgery, a 5 × 10 mm pedunculated polypoid mass attached to anterior mitral chorda was removed by simple excision, preserving mitral valve function.

Tamin et al. [10] evaluated the pathology and echocardiography database of Mayo Clinic (Rochester, MN, U.S.A.) for PFE and cardiac myxoma occurring between 1995 and 2010. This study showed that PFE is more common than cardiac myxoma, at a rate of approximately 2 : 1, for a rate of 1 PFE per every 1,100 echocardiograms; in more than 500 cases with a presumed PFE diagnosis, 185 patients had surgical tumour removal and 326 patients did not have surgical resection for different reasons. Furthermore, a significant association was found between clinically diagnosed PFE and neurologic events (NE); both mortality and risk of cerebrovascular accidents are greater in patients with PFE identified with echocardiography and not removed than with age- and sex-matched rates. In patients undergoing PFE surgical removal, median postoperative follow-up was 1.6 years showing a recurrent PFE in 3 cases (1.6%), at 1 year and 5 years, 95% and 86% of patients, respectively, were alive; overall risk of CVA was 2% at 1 year and 8% at 5 years; 10 CVA were found compared with 4.1 expected CVA on age- and sex-matched rates. In the conservative group, median follow-up was 1.7 years, and the CVA risk was 6% and 13% at 1 and 5 years, respectively, 29 CVA compared with 8 expected in the matched control population; 1-year and 5-year survival were, respectively, 87% and 67%. Histological PFE study showed surface thrombi may occur, and for this reason, it suggested long-term antiplatelet agents. The study concludes that in patients with echocardiographically suspected PFE who do not undergo surgical removal, rates of cerebrovascular accident and mortality are increased.

In a comprehensive analysis of 725 cases found in the literature, Gowda et al. [3] found 25 cases not operated but with adverse outcomes: of these, 12 patients had PFE-related death.

Sang-Hoon et al. [11] reported a probable PFE with a stalk on the septal leaflet of the tricuspid valve in a 25-year-old woman that was followed at two months intervals for 3 years: she was asymptomatic and the tricuspid mass size decreased. PFE is located in the high flow and high pressure left heart, the risk of embolism is higher than the one located in the right cardiac system.

Our reported case presents a special interest because in the reviewed literature it is the longest follow-up, 19 years, in a patient with an echocardiographic PFE diagnosis who did not undergo surgical removal of the tumour. It is well known that most PFE reported are incidental findings in asymptomatic patients but embolic complications may be life-threatening. Our patient refused the proposed surgical treatment and was followed up yearly.

We do believe that in symptomatic patients, surgical PFE located in the left heart should be removed but our analysis shows that further data in this issue is needed in asymptomatic patients, and surgical indication should be proposed considering carefully the location of the mass, size of the mass, morphology and other clinical characteristics, and the risk-benefit balance.

## Data Availability

Consent to publication form has been added for the use of data.
